# Angiotensin-Converting Enzyme Gene Insertion/Deletion Polymorphism and Small Vessel Cerebral Stroke in Indian Population

**DOI:** 10.1155/2014/305309

**Published:** 2014-01-12

**Authors:** Puttachandra Prabhakar, Tanima De, Dindagur Nagaraja, Rita Christopher

**Affiliations:** ^1^Department of Neurochemistry, National Institute of Mental Health and Neuro Sciences, Bangalore 560029, India; ^2^Department of Neurology, National Institute of Mental Health and Neuro Sciences, Bangalore 560029, India

## Abstract

*Background*. Hypertension is an established risk factor for small-vessel cerebral stroke and the renin-angiotensin system plays an important role in the maintenance of blood pressure. We aimed at evaluating the contribution of the angiotensin-converting enzyme (*ACE*) gene insertion/deletion (I/D) polymorphism to the risk of small-vessel stroke in south Indian population. *Materials and Methods*. We investigated 128 patients diagnosed with small-vessel stroke and 236 age, and gender-matched healthy controls. *ACE* I/D polymorphism was detected by polymerase chain reaction. *Results*. Hypertension was significantly more prevalent in the patient group and was associated with 6-fold increase in risk for stroke. *ACE* genotypes were in Hardy-Weinberg equilibrium in both patients and controls. Prevalence of DD, ID, and II genotypes in cases (34.4%, 43.7%, and 28%) did not differ significantly from controls (31.8%, 43.2%, and 25%). The polymorphism was not associated with small-vessel stroke (OR: 1.34; 95% CI: 0.52–1.55). However, diastolic blood pressure was associated with the *ACE* I/D genotypes in the patients. (DD; 90.2 ± 14.2> ID; 86.2 ± 11.9> II; 82.3 ± 7.8 mm Hg,  *P* = 0.047). *Conclusion*. Our study showed that hypertension, but not *ACE* I/D polymorphism, increased the risk of small-vessel stroke.

## 1. Introduction

Brain infarction due to ischemia in the perforating arteries which supply the brain white and deep grey matter nuclei, also known as lacunar infarction or lacunar stroke, accounts for 20% to 25% of all ischemic strokes [1]. Lacunar infarct forms part of the spectrum of cerebral small-vessel disease (SVD) which affects the brain diffusely and is the commonest vascular cause of cognitive impairment [2]. The prevalence rate of stroke in India varies across regions. While the prevalence is 5.4 per 1000 persons in the eastern part of India [3], southern India has reported a prevalence of 1.36 per 1000 persons [4]. The Indian Collaborative Acute Stroke Study (ICASS), a recent multicenter study conducted among 2162 admitted stroke patients across India, observed ischemic stroke in 77%, hemorrhagic stroke in 22%, and unspecified stroke in 1% of cases [5]. Data from a hospital-based stroke registry from south India shows that, of all ischemic stroke patients, 41%, 18%, 10%, 4%, and 27% were classified as large-artery atherosclerosis, lacunae, cardioembolism, other determined etiology, and undetermined etiology, respectively [6]. Hypertension, diabetes, and smoking were the common risk factors among all the subtypes [7], similar to other populations. Hypertension is considered the foremost risk factor for lacunar stroke [8]. Several lines of evidence from family and twin studies support the hypothesis that genetic factors may contribute to the pathogenesis of SVD and lacunar stroke [9].

The gene encoding angiotensin-converting enzyme (*ACE*), which converts angiotensin I to the vasoconstrictor angiotensin II and inactivates the vasodilator bradykinin, is considered to be an important candidate in cerebral SVD because of its role in blood pressure regulation, regulation of vascular endothelial function, and smooth muscle proliferation and tone [10]. The I/D polymorphism in intron 16 of *ACE* is one of the most frequently studied genetic variations across different populations for association with vascular diseases. Despite being an intronic variation, it accounts for approximately half of the observed variance in plasma levels of the *ACE* protein [10]. Previous studies on *ACE* I/D polymorphisms in lacunar strokes and other cardiovascular phenotypes have yielded conflicting results. The *ACE* D allele has been reported to be significantly associated with lacunar stroke in a few studies [8]. Few studies from western population have found no association [11, 12]. Since the role of *ACE* I/D polymorphisms in the pathogenesis of ischemic stroke due to cerebral SVD is controversial, our aim was to evaluate the association of this polymorphism with small-vessel stroke in south Indian population.

## 2. Materials and Methods

The study group comprised of 128 first-ever stroke patients, aged between 40 and 65 years, seen at either the emergency department or the neurology outpatient services, within a week after the onset of symptoms of the classic lacunar syndromes (pure motor hemiparesis, pure sensory stroke, sensorimotor stroke, ataxic hemiparesis, and dysarthria-clumsy hand syndrome), and diagnosed with stroke classed as “small-vessel” by the Trial of ORG 10172 in Acute Stroke Treatment (TOAST) classification [13]. Small-vessel occlusion or lacunar infarct was defined as an acute classic lacunar syndrome with an MRI showing a subcortical hemispheric ischemic lesion with a diameter of less than 15 mm. Patients with potential cardiac sources of embolism or stenosis >50% in an ipsilateral brain-supplying artery were excluded. Similarly, patients with other subtypes of ischemic stroke, transient ischemic attack, intracerebral hemorrhage, subarachnoid hemorrhage, arterial dissection, brain tumors, cerebrovascular malformation, neuroinfections, severe systemic diseases, and hepatic or renal dysfunction were excluded. 236 age, and gender-matched healthy volunteers served as the control group. Both patients and controls were from the same geographical location.

Informed consents were obtained from both groups. Baseline demographic data, history of conventional vascular risk factors and previous vascular events was obtained. Hypertension were defined as systolic blood pressure (SBP) of >140 mm Hg/diastolic blood pressure (DBP) >90 mm Hg/treatment with antihypertensive drugs. Diabetes mellitus was defined as venous plasma glucose concentration of ≥7.0 mmol/L after an overnight fast and/or ≥11.1 mmol/L 2 hours after a meal or the use of insulin or oral hypoglycemic agents.

**Figure 1:**
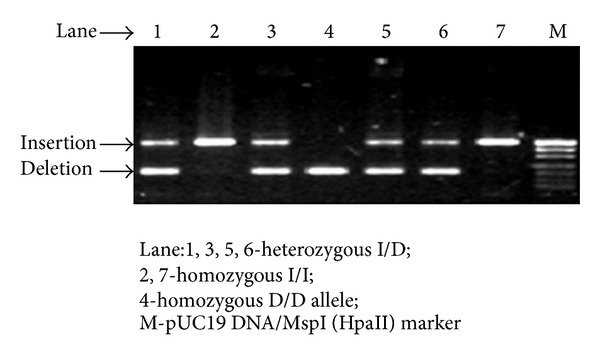
Representative band patterns of *ACE* I/D polymorphism analyzed using agarose gel electrophoresis.

Venous blood samples were collected and genomic DNA isolated from nucleated blood cells by Triton X-100 salt-precipitation method. Genotyping for *ACE* I/D polymorphism was done by polymerase chain (PCR) reaction as described by Rigat et al. [14]. After completion of PCR cycles the product was analyzed in 1.8% agarose gel (Sigma-Aldrich, Bangalore, India) and was captured in a gel documentation system (Bio-Rad, USA). In *ACE* DD genotype, a single band of 191 bp and, in *ACE* II genotype, a band of 490 bp were observed. Both bands were observed in the heterozygous condition (Figure 1).

Statistical data analysis was performed using IBM SPSS statistics 17.0 (IBM Corporation, NY, USA) and Graph Pad prism version 5.0.1 (Graph Pad Software, Inc. La Jolla, USA). Differences in baseline characteristics between patients and controls were assessed by the *χ* ^2^ test for categorical variables and the *t*-test for continuous parameters. The frequencies of the alleles and genotypes were compared between patient and control groups by the *χ* ^2^ test. Hardy-Weinberg equilibrium (HWE) for each genotype polymorphism was performed for control subjects by the *χ* ^2^ test. Univariate odds ratio (OR) and 95% confidence interval (CI) were estimated and a multiple logistic regression analysis was used to adjust the OR for age and gender.

## 3. Results

The average ages of controls and cases were 49.1 ± 11.6 and 51.4 ± 14.0 years, respectively. The demographic characteristics of the two groups are shown in Table 1. Non genetic variables such as smoking, and hypertension were found to be more prevalent in patients compared to controls. The mean SBP (*P* = 0.001) and DBP (*P* = 0.001) were significantly elevated in cases compared to controls. Hypertension was associated with a 6-fold increase in risk for small-vessel stroke (OR: 5.87; 95% CI: 3.46–9.96, *P* = 0.002).

The genotype frequencies of II, ID, and DD were 31.8%, 43.2%, 25.0% in controls and 34.4%, 43.7%, and 21.9% and in cases, respectively (Table 1). Genotype distribution complied with Hardy-Weinberg equilibrium (*P* > 0.05). I allele frequencies in controls and cases were 0.53 and 0.58, respectively. As shown in Table 1, logistic regression analysis after adjustment for covariates did not detect any association between the polymorphism and small-vessel stroke, when either the dominant (ID+DD versus II) or recessive (ID+II versus DD) models were used. The DBP was elevated in cases with ID and DD genotypes (*P* = 0.047) (Table 2).

## 4. Discussion

*ACE* enzyme is an attractive candidate to play a role in vascular diseases. The I/D polymorphism in *ACE* gene has been reported to account for 47% of the total phenotypic variance of serum *ACE* that causes renin-dependent hypertension [10]. Several studies have demonstrated the importance of *ACE* I/D polymorphisms in the pathogenesis of hypertension[8].

The role of hypertension as an independent risk factor for lacunar stroke has been reiterated in our study which showed that hypertension increased the risk 5.87-fold (OR: 5.87; 95% CI: 3.46–9.96, *P* = 0.002). However, no significant association of *ACE* polymorphism with lacunar stroke was observed in the present study. Our findings are supported by previous studies in Polish and Turkish populations which reported no significant association of *ACE* I/D polymorphism with small-vessel stroke [11, 12]. Similarly, Munshi et al., found no association in 28 small artery occlusion patients [15]. On the contrary, in an early report on 18 cases of lacunar stroke, Markus et al. observed that the presence of *ACE* DD was associated with 4.4-fold increase in the risk for the disease (OR: 4.4, 95% CI: 1.45–12.60) [8]. Similarly, Szolnoki and coworkers found 3.4-fold increase in the risk for small-vessel ischemic stroke in the Hungarian population with *ACE* DD (OR: 3.44, 95% CI, 1.9–6.24; *P* < 0.0005) [16]. Insufficient power in some studies and the interaction with other genes or environmental factors are possible explanations for the contradictory findings.

In a recent large meta-analysis of 50 studies relating the *ACE* I/D polymorphism to the risk of ischemic stroke, Zhang and coworkers found that DD homozygote carriers had a 37% higher risk of ischemic stroke when compared with the homozygotes II and heterozygote ID (OR = 1.37, 95% CI: 1.22–1.53). Subgroup analyses showed that this higher risk was more evident among Asians, hospital-based studies, and small-vessel stroke [17]. They suggested that genetic risk factors for different subtypes are likely different, supporting the view that they are pathologically distinct entities, with small-vessel stroke having a greater genetic liability compared to large vessel disease.

## 5. Conclusion

Identifying common gene variants that might increase the risk of small-vessel ischemic stroke is of public health importance because it could help to recognize and treat sub populations that might be at an increased risk for the disease. Our study demonstrates that hypertension, but not the *ACE* I/D polymorphism, is associated with a risk of small-vessel stroke in south Indian population.

## Figures and Tables

**Table 1:** The demographic characteristics and *ACE* I/D genotype distributions of the study groups.

Characteristics	Controls, *n* = 236 (%)	Cases *n* = 128 (%)
Age, years (mean ± SD)	49.1 ± 11.6	51.4 ± 14.0^ns^
Male/female (%)	142/94 (60.1/39.9)	81/47 (63.6/36.4)^ns^
SBP (mm Hg)	124.03 ± 10.83	137.05 ± 22.03^†^
DBP (mm Hg)	81.58 ± 9.17	85.93 ± 11.65^†^
Smokers (%)	22 (9.5)	31 (24.4)^†^
Alcohol use (%)	19 (8.2)	19 (14.7)^ns^
HT (%)	30 (12.7)	62 (48.4)^†^
DM (%)	16 (6.9)	10 (7.7)^ns^

*ACE* I/D genotype	Control *n* = 236 (%)	Case *n* = 128 (%)

II	75 (31.8)	44 (34.4)
ID	102 (43.2)	56 (43.7)
DD	59 (25.0)	28 (21.9)

OR at 95% CI	UOR	AOR^§^

ID versus II	0.93 (0.57–1.53)^ns^	1.12 (0.49–1.42)^ns^
DD versus II	0.81 (0.45–1.45)^ns^	1.23 (0.51–1.91)^ns^
ID + DD versus II (dominant)	0.89 (0.56–1.41)^ns^	0.92 (0.29–1.22)^ns^
ID + II versus DD (recessive)	1.19 (0.71–1.98)^ns^	1.34 (0.52–1.55)^ns^

*ACE*: angiotensin-converting enzyme; HT: hypertensive; DM: diabetic; OR: odds ratio; UOR: unadjusted odds ratio; AOR: adjusted odds ratio.

^†^ *P* < 0.002; ^ns^ *P* > 0.2; ^§^with age and gender.

**Table 2:** Association of systolic and diastolic blood pressure with *ACE* I/D polymorphism.

Genotype		II (%)	ID (%)	DD (%)	*P* value
SBP	Controls	126 ± 12.3 (29.7)	121.8 ± 9.3 (48.6)	126.3 ± 11.4 (21.6)	0.115
Mean ± SD (mm)	Cases	132.2 ± 14.9 (32.9)	138.6 ± 26.2 (41.7)	140.9 ± 22.2 (25.3)	0.316

DBP	Controls	82.8 ± 12.1 (29.7)	80.8 ± 7.4 (48.6)	81.5 ± 8.3 (21.6)	0.603
Mean ± SD (mm)	Cases	82.3 ± 7.8 (32.9)	86.2 ± 11.9 (41.7)	90.2 ± 14.2 (25.3)	0.047*

SBP: systolic blood pressure; DBP: diastolic blood pressure.

*Significant.
